# The effect of Huntington’s disease on cognitive and physical motivation

**DOI:** 10.1093/brain/awae023

**Published:** 2024-01-24

**Authors:** Kelly J Atkins, Sophie C Andrews, Julie C Stout, Trevor T J Chong

**Affiliations:** Turner Institute for Brain and Mental Health, School of Psychological Sciences, Monash University, Melbourne, Victoria 3800, Australia; Turner Institute for Brain and Mental Health, School of Psychological Sciences, Monash University, Melbourne, Victoria 3800, Australia; Thompson Institute, University of the Sunshine Coast, Queensland 4575, Australia; Turner Institute for Brain and Mental Health, School of Psychological Sciences, Monash University, Melbourne, Victoria 3800, Australia; Turner Institute for Brain and Mental Health, School of Psychological Sciences, Monash University, Melbourne, Victoria 3800, Australia; Department of Neurology, Alfred Health, Melbourne, Victoria 3004, Australia; Department of Clinical Neurosciences, St Vincent’s Hospital, Melbourne, Victoria 3065, Australia

**Keywords:** Huntington’s disease, motivation, effort, apathy, decision-making

## Abstract

Apathy is one of the most common neuropsychiatric features of Huntington’s disease. A hallmark of apathy is diminished goal-directed behaviour, which is characterized by a lower motivation to engage in cognitively or physically effortful actions. However, it remains unclear whether this reduction in goal-directed behaviour is driven primarily by a motivational deficit and/or is secondary to the progressive cognitive and physical deficits that accompany more advanced disease.

We addressed this question by testing 17 individuals with manifest Huntington’s disease and 22 age-matched controls on an effort-based decision-making paradigm. Participants were first trained on separate cognitively and physically effortful tasks and provided explicit feedback about their performance. Next, they chose on separate trials how much effort they were willing to exert in each domain in return for varying reward. At the conclusion of the experiment, participants were asked to rate their subjective perception of task load.

In the cognitive task, the Huntington’s disease group were more averse to cognitive effort than controls. Although the Huntington’s disease group were more impaired than controls on the task itself, their greater aversion to cognitive effort persisted even after controlling for task performance. This suggests that the lower levels of cognitive motivation in the Huntington’s disease group relative to controls was most likely driven by a primary motivational deficit. In contrast, both groups expressed a similar preference for physical effort. Importantly, the similar levels of physical motivation across both groups occurred even though participants with Huntington’s disease performed objectively worse than controls on the physical effort task, and were aware of their performance through explicit feedback on each trial. This indicates that the seemingly preserved level of physical motivation in Huntington’s disease was driven by a willingness to engage in physically effortful actions despite a reduced capacity to do so. Finally, the Huntington’s disease group provided higher ratings of subjective task demand than controls for the cognitive (but not physical) effort task and when assessing the mental (but not the physical) load of each task.

Together, these results revealed a dissociation in cognitive and physical motivation deficits between Huntington’s disease and controls, which were accompanied by differences in how effort was subjectively perceived by the two groups. This highlights that motivation is the final manifestation of a complex set of mechanisms involved in effort processing, which are separable across different domains of behaviour. These findings have important clinical implications for the day-to-day management of apathy in Huntington’s disease.

## Teaser

Atkins *et al*. report that people with Huntington’s disease are less willing to exert cognitive effort than controls. However, they are similarly willing to exert physical effort, despite an impaired ability to do so. This suggests that cognitive and physical motivation are driven by separable domain-specific mechanisms.

## Introduction

Apathy is one of the most common and debilitating psychiatric features of Huntington’s disease (HD).^1,2^ A central feature of apathy is diminished goal-directed behaviour, which has recently been operationalized as a lower motivation to engage in behaviour that is cognitively or physically effortful.^3-6^ The prevalence of apathy in HD increases with disease progression and parallels the development of physical and cognitive disability.^7-10^ Consequently, apathy is typically accompanied by a multitude of other impairments, including involuntary movements, disrupted sensorimotor processing^11,12^ and cognitive impairment.^13,14^ This therefore raises the question of whether motivation in HD is reduced even after accounting for an individual’s cognitive or motor deficits and, if so, to what extent this lower motivation is unique to particular domains (e.g. cognitive, physical).

Although several definitions of apathy exist, all such definitions require that the reduction in goal-directed activity cannot be secondary to other causes, such as intellectual, physical or motor disabilities.^15,16^ In practice, however, these can be difficult to exclude because many patients with apathy also experience concurrent cognitive impairment or physical disability.^7-10^ In HD, these disabilities are known to result, not only in impaired cognitive and physical performance, but also impaired sensorimotor processing. This may in turn impair one’s ability to accurately perceive the demands of a task or estimate the extent of one’s disability (as in anosognosia^17,18^). Excluding these issues as a primary cause of reduced goal-directed behaviour is especially challenging given that the increase in prevalence of motivational deficits parallels the increase in motor and cognitive disability. Clinical questionnaires and inventories often focus on the final manifestations of goal-directed behaviour and do not consider the underlying reasons for any such deficits.^19^ Consequently, the impression of whether cognitive or physical disability could be confounding an assessment of apathy is often left to clinical judgement.

Recent experimental paradigms have the potential to demonstrate with greater precision the extent to which a primary cognitive or motor impairment may be influencing goal-directed actions. A common approach has been to quantify apathy in terms of the amount of effort that people are willing to invest in return for reward.^5,6,20,21^ Such paradigms typically expose participants to the amount of effort required to perform increasingly demanding levels of a task before then asking them to choose the level of effort they are willing to engage in return for varying levels of rewards.^3,22^ These tasks have the potential to analyse the willingness of individuals to invest effort while controlling for their capacity to do so. This in turn can disentangle a motivational deficit (the willingness to overcome an aversive action) from a primary cognitive or motor impairment. These objective data can be coupled with subjective ratings of perceived effort to determine how the experience of effort is translated into decisions to avoid it.

An important feature of the diminished goal-directed behaviour in apathy is that it can be experienced across multiple domains.^15,16,19^ A common distinction is between the willingness of people to engage in goal-directed cognitive versus physical activity.^23-25^ Recent work has suggested a core network underlying motivation across both domains, which is likely centred on the striatum and prefrontal cortex.^24^ For example, patients with Parkinson’s disease often experience deficits in both cognitive and physical motivation.^26,27^ However, the mechanisms underlying goal-directed behaviour are also at least partly dissociable. For example, people with pre-manifest HD have lower cognitive motivation than control subjects, but are similarly physically motivated.^28^ This dissociation occurred despite pre-manifest and control groups being closely matched both in their cognitive and physical abilities. However, what remains unclear is whether this dissociation persists in more advanced stages of the disease, particularly given that HD progressively affects both cognitive and physical function, and at different rates.

The broad goal of this study was to determine how manifest HD affects the willingness to engage in cognitively or physically effortful behaviour. Importantly, we aimed to determine whether any such differences in behaviour are driven primarily by a motivational deficit, even after accounting for the capacity of individuals to perform the tasks themselves. Given the increasing cognitive and physical disability in HD, we expected the HD cohort to have lower motivation across both domains relative to controls, although a critical question is whether such deficits persist even after accounting for other aspects of the disease that affect task performance.

We tested 17 patients with manifest HD and compared their performance to 22 matched control subjects. Participants were first trained on separate cognitively and physically effortful tasks, before then deciding how much effort they were willing to invest in each domain in return for various rewards. Importantly, in our analyses, we accounted for the degree to which participants’ choices were influenced by their capacity to perform each task, which allowed us to distinguish lower goal-directed behaviour due primarily to a motivational deficit versus reduced performance capacity. At the conclusion of the experiment, we asked participants to rate the subjective task load of each level of the cognitive and physical effort tasks, which allowed us to determine how the subjective experience of effort is translated into choice behaviour.

## Materials and methods

### Participants

We recruited 17 patients with HD and compared their performance to 22 gene-negative controls, matched for age and gender (Table 1). Exclusion criteria included a history of neurological disease (other than HD, in the case of the HD group), major traumatic brain injury, cerebrovascular accident or substance abuse. Patients with HD had between 41 and 54 CAG repeat expansions in the huntingtin gene and were, on average, in Stage 2 disease according to the Total Functional Capacity Scale on the Unified Huntington Disease Rating Scale (UHDRS).^34-36^ Participants were recruited from our internal research database, the Calvary Bethlehem Hospital in Melbourne, and the wider community. The HD cohort comprised an entirely independent set of individuals to those recruited for an earlier study we reported on pre-manifest disease.^28^ The study was approved by the Monash University Human Research Committee and all participants provided informed consent in accordance with the Declaration of Helsinki.

**Table 1:** Summary of participant demographics

	Healthy controls	Huntington’s disease	Group difference
*n*	22	17	N/A
Age, years	50.9 (11.2) [28–65]	53.3 (10.3) [28–65]	*P =* 0.49
Gender, male:female	12:10	12:5	*P =* 0.31
Apathy Evaluation Scale^a^	10.8 (5.89)	15.5 (9.85)	*P =* 0.09
Dimensional Apathy Scale, Total^b^	22.7 (7.12)	36.0 (10.8)	*P <* 0.001*
Executive	5.71 (3.77)	12.9 (5.94)	*P <* 0.001*
Initiation	8.62 (3.07)	12.1 (4.59)	*P =* 0.01*
Emotional	8.38 (3.02)	11.1 (3.72)	*P =* 0.03*
Hospital Anxiety and Depression Scale^c^			
Anxiety	5.33 (3.44)	5.18 (3.34)	*P =* 0.89
Depression	3.10 (2.68)	5.71 (4.18)	*P =* 0.03*
Montreal Cognitive Assessment^d^	27.0 (2.01)	23.6 (2.83)	*P <* 0.001*
Hopkins Verbal Learning Test Revised			
Total Recall	26.9 (3.10)	17.0 (4.54)	*P <* 0.001*
Delayed Recall	9.6 (1.93)	4.71 (2.73)	*P <* 0.001*
Discrimination Index	11.1 (1.97)	8.13 (3.26)	*P =* 0.004*
Symbol Digit Modalities Test	58.8 (12.6)	26.6 (9.27)	*P <* 0.001*
Tapping (mean intertap interval, ms)^29^			
Speeded	195.1 (34.2)	347.4 (85.2)	*P <* 0.001*
Paced	39.0 (13.5)	216.7 (243.8)	*P* = 0.01*
CAG repeats	N/A	43.4 (3.02) [41–54]	N/A
Total functional capacity			
Self-rated	N/A	8.59 (2.27) [6–13]	N/A
Informant	N/A	8.64 (2.20) [5–13]	N/A
Disease Burden Score^30^	N/A	399.8 (85.5) [192.5–518]	N/A

Data are presented as means (SD) [range]. N/A = not applicable.

^a^Range from 18 to 72. Proposed cut-off scores for apathy in Huntington’s disease are >40 (Naarding *et al*.^31^) and >41 (Skvortsova *et al*.^32^).

^b^Proposed cut-off score for apathy ≥38.^19^

^c^Subscale cut-off of 8.^33^

^d^Cut-off <26.

^*^Statistically significant, *P* < 0.05.

We assessed cognition using several performance-based measures, including: a standard cognitive screening tool (the Montreal Cognitive Assessment, MoCA), as well as neuropsychological tests of episodic memory (Hopkins Verbal Learning Test-Revised, HVLT-R) and attention/psychomotor speed (Symbol Digit Modalities Test, SDMT). Motor performance was quantified on tests of speeded and paced tapping, which have been shown to be sensitive to the motor deficits in HD.^29^ We used the self-reported Hospital Anxiety and Depression Scale (HADS)^37^ to measure depressive symptoms. Apathy was assessed using the Apathy Evaluation Scale (AES), which provides a total apathy score^38^ and the Dimensional Apathy Scale (DAS), which separates apathy into ‘Executive’, ‘Initiation’ and ‘Emotional’ subtypes.^39^ Both the AES and DAS have been validated in HD.^19,31,32,40^

### Procedure

**Figure 1:**
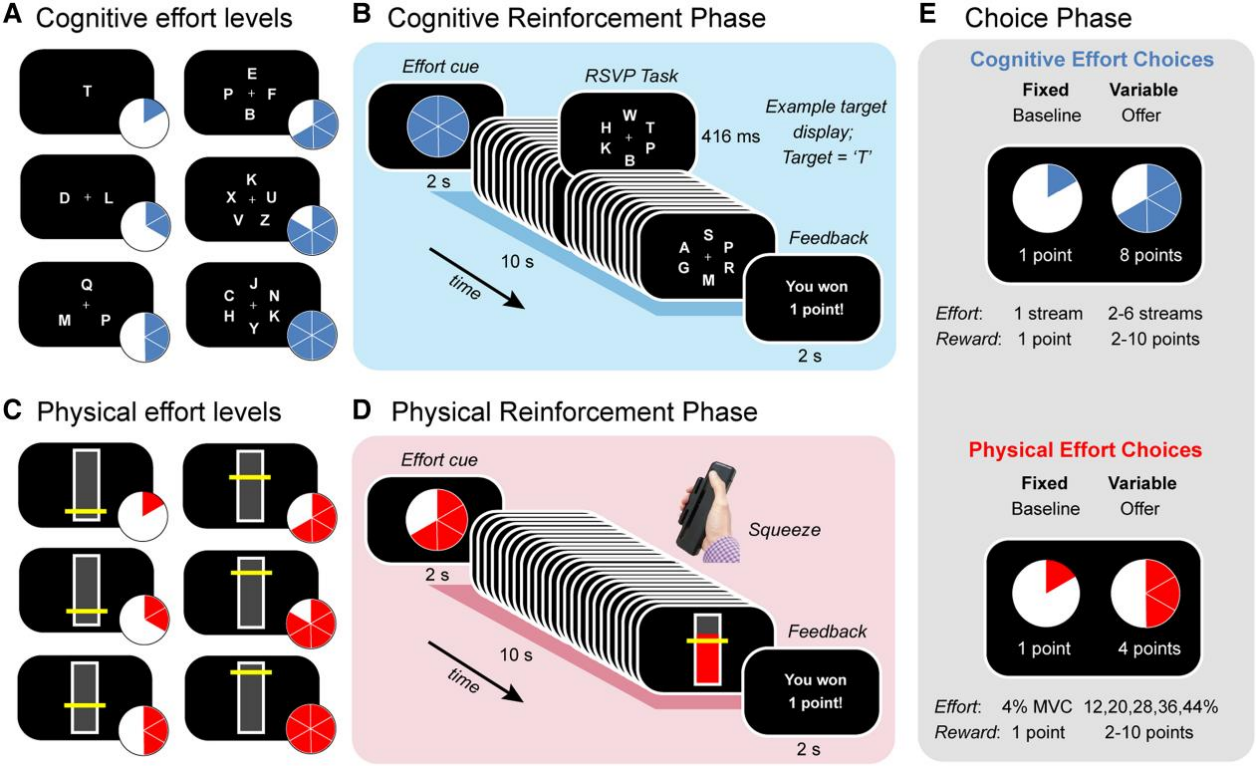
**Task design.** Participants were first trained on (**A** and **B**) a cognitively effortful task and (**C** and **D**) a physically effortful task, before (**E**) indicating their preference for investing effort for reward. (**A**) The cognitive effort task required participants to monitor one to six rapid serial visual presentation (RSVP) streams for a target letter (‘T’). (**B**) Each trial began with a blue pie chart indicating the number of streams they had to monitor on that trial (here, six streams, i.e. Effort Level 6). After completing each effort level, participants received feedback on their performance. (**C**) The physical effort task required participants to sustain variable amounts of force on a hand-held dynamometer, with the target levels of force defined as a function of each individual’s maximum voluntary contraction (MVC; 4, 12, 20, 28, 36, 44%). (**D**) Each trial began with a red pie chart indicating the amount of force they had to apply on that trial. Trial durations were identical to those for the cognitive effort task (10 s). At the conclusion of each trial, participants received feedback on their performance. (**E**) The choice phase required participants to decide how much effort they were willing to invest for reward. The choice was always between a fixed baseline option [the lowest level of effort for the lowest reward (one point)] and a variable high-effort/high-reward offer (higher levels of effort; rewards of 2 to 10 points). Separate choices were made for cognitive and physical effort.

Each testing session began with administration of a cognitive screening test (MoCA), which was followed by the effort-based decision-making task and finally our neuropsychological and tapping tests. The decision-making task was identical to that described in an earlier study in pre-manifest HD.^28^ This task was divided into three phases (Fig.1). The first two (‘Reinforcement’) phases involved training participants on both a cognitively effortful task (Fig. 1A and B) and a physically effortful task (Fig. 1C and D), in counterbalanced order. Within each task, we parametrically varied demands in the target domain (e.g. cognitive), while keeping those in the other (e.g. physical) constant. The reinforcement phases were followed by a final ‘Choice’ phase, during which participants were asked to choose between a fixed low-effort/low-reward option and a variable high-effort/high-reward offer (Fig. 1E). These choices allowed us to quantify the willingness of participants to exert distinct types of effort.

#### Reinforcement phase

##### Cognitive effort task

The cognitive effort task applied a rapid serial visual presentation (RSVP) paradigm, in which participants monitored a series of rapidly changing letters for the target letter, ‘T’ (Fig. 1A and B). We varied cognitive load by increasing the number of streams from one to six. In the least effortful condition (Level 1), a single letter stream was presented at fixation. In the more effortful conditions (Levels 2–6), we manipulated cognitive effort by increasing the number of streams to which participants had to attend (between two and six). Each stream was positioned equidistantly around fixation. The target letter could appear randomly in any stream and the timing of the target stimuli was pseudorandom, such that they could not appear in consecutive stimulus frames. Each effort level comprised 24 stimulus frames, each of which lasted 416 ms, for a total trial duration of 10 s.

Each trial began with a blue pie chart, which cued the level of cognitive effort to follow. After performing that level, they received feedback about their success. They were rewarded with one point for each trial they performed above a threshold level of performance (more than one hit; fewer than three false alarms); otherwise they were not rewarded. Participants were instructed to maximize the number of points won. Participants completed two blocks of 30 trials, with an opportunity to rest after each block (i.e. a total of 10 trials per effort level, randomly allocated). These experimental blocks were preceded by a practice block of 12 trials (two per effort level). Responses were registered on a Cedrus button box and the task was implemented on Presentation software (Neurobehavioral Systems).

##### Physical effort task

In the physical effort task, participants were required to exert one of six levels of force on a hand-held dynamometer (SS25LA, BIOPAC systems) using their dominant hand (Fig. 1C and D). At the beginning of the experiment, we defined participants’ maximum voluntary contraction (MVC) as the maximum of three consecutive squeezes. To standardize effort requirements across participants, we defined the target effort levels for each participant as a function of their own MVC (4, 12, 20, 28, 36, 44%). Target levels were visually depicted as a horizontal yellow line on a vertical bar and participants received real-time visual feedback of their applied force.

Each trial in the physical effort task commenced with a red pie chart, which cued the level of physical effort required on that trial. Participants then had to initiate their contraction and maintain it above the required effort level for at least 50% of the total trial duration (i.e. ≥ 5 of 10 s) to be successfully rewarded. Importantly, the physical effort task was identical to the cognitive effort task in terms of the trial durations (10 s per effort level); number of trials per effort level (10 trials per effort level); and overall block structure (two blocks of 30 trials). The physical effort task was implemented on Psychtoolbox (http://psychtoolbox.org) running in MATLAB (Mathworks, USA).

#### Choice phase

Next, participants undertook the critical choice phase, which allowed us to measure the key outcome of interest—the willingness to exert cognitive and physical effort. In this phase, participants revealed their preference between a fixed, low-effort/low-reward baseline option, and a variable, high-effort/high-reward offer. The fixed baseline option was always the option to exert the lowest amount of effort for the lowest reward (one point). In contrast, the variable offer was the option to exert a higher amount of effort (Levels 2–6) for a greater reward (2–10 points). To separate individuals’ cognitive and physical motivation, each choice was always made between two options in the same domain. We sampled the entire effort-reward space evenly and randomly across both domains over a total of 150 trials (six trials per effort-reward condition). Participants made their selection with a button press and trials were self-paced. To reduce the impact of fatigue on subsequent decision-making, participants were not required to execute their choices, but simply indicate their preferred option. They were explicitly told that their decisions were hypothetical, in that points did not alter remuneration, but that they should select the option that was most preferable to them. This protocol is consistent with previous studies.^23,27,41,42^

#### Assessment of subjective task load

To confirm that our tasks were effective in manipulating the subjective perception of effort in the cognitive and physical domains, participants were required to complete the NASA Task Load Index.^43^ We focused on the subscales in which participants rated their subjective experience of mental demand (‘How mentally demanding was the task?’) and physical demand (‘How physically demanding was the task?’).^44^ Results for the remaining subscales are presented in the Supplementary material. Participants provided responses to each question on a 21-point scale (−10 to +10), with higher scores indicating higher perceived task load. Participants rated the mental and physical demand of each effort level on both the cognitive and physical effort tasks.

## Results

**Figure 2:**
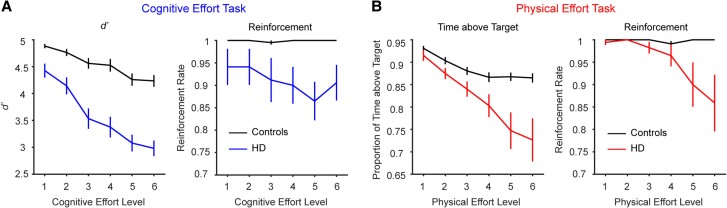
**Performance in the (A) cognitive and (B) physical effort tasks (mean ± 1 SEM).** (**A**) In the cognitive effort task, decrements in target detection sensitivity (*left*) and reinforcement rates (*right*) with increasing effort were more pronounced in the Huntington’s disease (HD) group (in blue) than controls (in black). (**B**) A similar pattern of results was found in the physical effort task. The proportion of time participants were able to maintain their force above the target level decreased with effort (*left*) and this decrement was steeper in the HD group (in red) than controls (in black). This was reflected in the reinforcement rates of each group (*right*). SEM = standard error of the mean.

First, we present data from the reinforcement phases to determine the differences in the capacity of each group to perform each of the cognitive and physical effort tasks (Fig. 2).

### Reinforcement phase

#### Cognitive effort task

In the cognitive effort task, performance was quantified as target detection sensitivity, *d’* [*Z*(Hits) − *Z*(False alarms)]. We used a repeated measures ANOVA to determine the effect of Group (controls, HD) on *d’* as a function of cognitive effort (1–6). Both main effects were significant and qualified by a significant two-way interaction [Group, *F*(1,37) = 45.8, *P <* 0.001; Effort, *F*(3.62,133.9) = 46.2, *P <* 0.001; interaction, *F*(3.62,133.9) = 7.16, *P <* 0.001]. Decomposing this interaction with Bonferroni-corrected pairwise comparisons revealed that the decrement in *d’* with increasing effort was steeper in the HD group relative to controls. For example, relative to the baseline effort level (Level 1), a significant drop in *d’* was only noted in controls at Level 5, but was already significant in the HD group by Level 3.

Next, we considered the capacity of each group to perform each task to the threshold required for reward. We performed the analogous ANOVA on the proportion of trials that individuals were successfully rewarded at each effort level (i.e. reinforcement rate). This again revealed a significant interaction [Group, *F*(1,37) = 6.3, *P* = 0.02; Effort, *F*(2.87,106.3) = 4.88, *P* = 0.004; interaction, *F*(2.87,106.3) = 4.90, *P* = 0.004], such that reinforcement rates were significantly lower in HD versus controls at effort Levels 4–6 (Levels 1–2, *P* > 0.10; Level 3, *P* = 0.06; Levels 4–6, *P* ≤ 0.009). Together, these results indicate that performance in the cognitive effort task was more impaired in HD relative to controls.

#### Physical effort task

We performed the analogous set of analyses on the physical effort task. We operationalized performance as the proportion of time that individuals were able to sustain their force above the target force level. The analogous Group × Physical effort level ANOVA on this metric again revealed that both main effects and their two-way interaction were significant [Group, *F*(1,37) = 12.4, *P =* 0.001; Effort, *F*(1.51,55.9) = 20.0, *P <* 0.001; interaction, *F*(1.51,55.9) = 5.34, *P =* 0.013]. Decomposing this interaction revealed that control performance decreased between Levels 1 and 4 (*P* ≤ 0.002), but plateaued thereafter. In contrast, performance in the HD group continued to decline between Level 1 and all higher levels of effort (*P* < 0.001).

The analyses on reinforcement rates revealed a similar two-way interaction [Group, *F*(1,37) = 6.71, *P =* 0.014; Effort, *F*(1.43,53.0) = 4.74, *P =* 0.022; interaction, *F*(1.43,53.0) = 4.88, *P =* 0.020]. Similar to the preceding analyses on cognitive effort, reinforcement rates were significantly lower in HD versus controls at the higher levels of effort (Levels 5–6, *P* ≤ 0.026; otherwise *P* > 0.12). Together, data from the reinforcement phase suggest that the capacity of the HD group to perform both the cognitive and physical effort tasks was impaired relative to controls, with the HD group in general demonstrating steeper decrements in performance relative to controls.

### Choice phase

**Figure 3:**
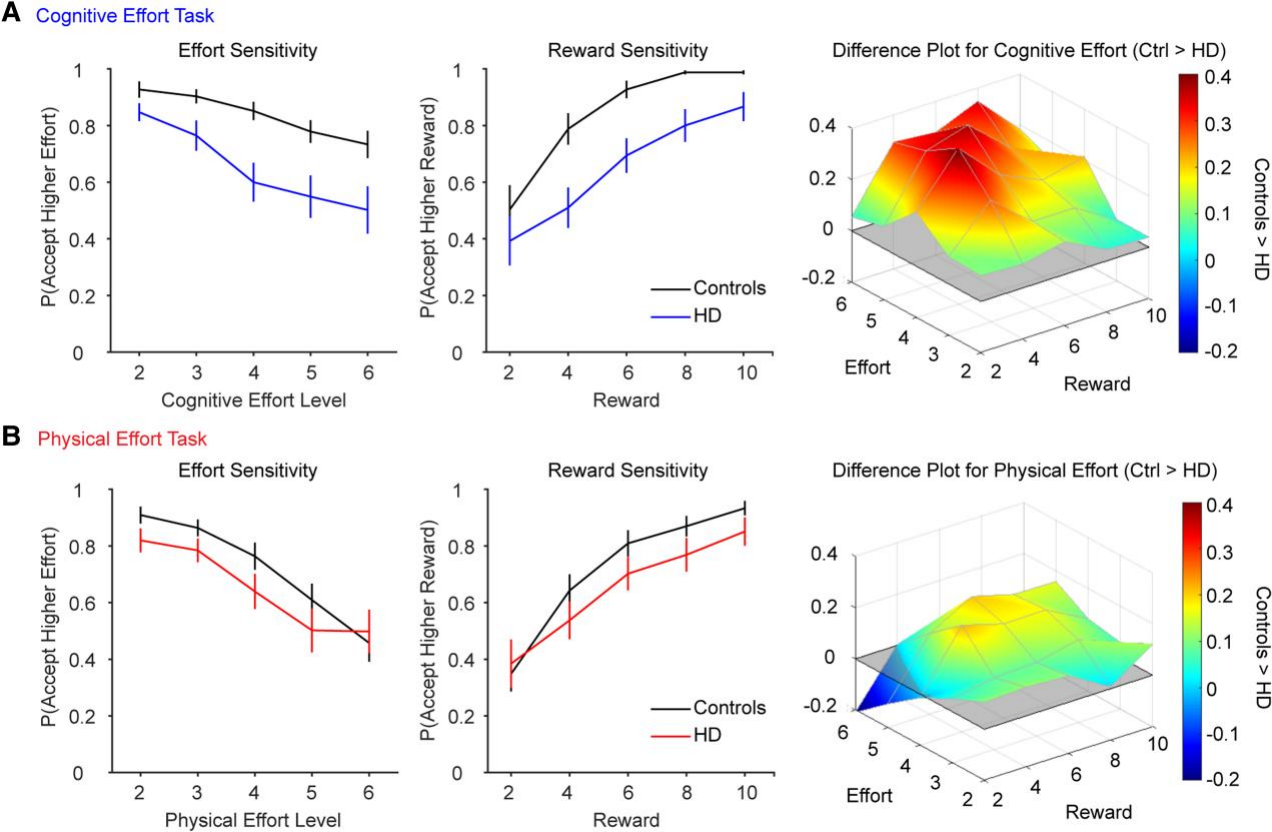
**Choices in the (A) cognitive and (B) physical effort tasks (mean ± 1 SEM).** Acceptance rates for the higher-effort/higher-reward offer are plotted as a function of effort (*left column*) and reward (*middle column*). Difference plots illustrate choice differences between the Huntington’s disease (HD) and control groups across the two-dimensional effort-reward space (*right column*). Red indicates greater motivation in controls than the HD group. (**A**) For cognitive effort-based choices, the HD group were less willing than controls to accept the higher-effort/higher-reward offers. (**B**) For the physical effort-based choices, group differences were less pronounced. SEM = standard error of the mean.

The critical question in this study was whether the HD group differed from controls in their willingness to exert cognitive or physical effort for reward (Fig. 3). An important consideration when examining effort-based decisions is to distinguish between the willingness of an individual to embark on an effortful action (i.e. their level of motivation) from their capacity to perform that action in the first place (i.e. difficulty).^3^ Given that the control and HD groups differed in their capacity to perform both tasks, it was critical to ensure that any group differences in motivation were driven by the perceived aversiveness of effort, rather than a reduced capacity to exert effort in the first place. We therefore analysed the data with a generalized linear mixed-effects model, which was capable of assessing all variables of interest on every trial in a single model.

In this model, choice was the outcome variable (baseline, offer) and we modelled the full range of simple effects and interactions involving the fixed effects of Group (control, HD), Domain (cognitive, physical), Effort (Levels 2–6) and Reward (Levels 2–6). To control for each participant’s capacity to perform each effort level, we included performance of the more effortful option on each trial as an additional fixed effect. An advantage of this approach is that it allowed us to precisely control for the effect of performance (i.e. difficulty), at each level of both tasks, on choice behaviour for every participant. Performance was quantified as *d’* for the cognitive effort task and time-above-target for the physical effort task, and was normalized separately within each domain. All within-subjects factors were fit as random slopes to each participant (Domain, Effort, Reward, Performance/Reinforcement Rate). We implemented the model using the *lme4* package implemented in R, using a binomial link function and bound optimization by quadratic approximation^45^ and we decomposed any significant interactions with the *phia* package.

Importantly, the effects of Group and Domain were significant and qualified by a significant Group × Domain interaction (Group, β = −4.30 ± 1.18, *Z =* −3.66, *P =* 0.0003; Domain, β = −3.52 ± 0.86, *Z =* −4.11, *P <* 0.001; Group × Domain, β = 3.02 ± 1.16, *Z* = 2.61, *P =* 0.009; Supplementary Table 1). Decomposing this interaction revealed that the HD group was overall significantly less willing than controls to exert effort in the cognitive domain (χ^2^ = 13.4, *P <* 0.001). However, in the physical domain, motivation was not significantly lower in the HD group relative to controls (χ^2^ = 2.50, *P =* 0.11).

The only other significant interactions involving Group were independent of Domain. There was a significant Group × Effort interaction (Group × Effort, β = 1.41 ± 0.49, *Z =* 2.90, *P =* 0.004; Effort, β = −2.63 ± 0.40, *Z =* −6.54, *P <* 0.001), which indicated that the reduction in motivation with increasing effort was greater in the HD group relative to controls (i.e. the former had a steeper effort discounting gradient; χ^2^ = 9.38, *P =* 0.002). Similarly, there was a significant Group × Reward interaction (Group × Reward, β = −2.29 ± 0.73, *Z =* −3.12, *P =* 0.002; Reward, β = 4.04 ± 0.55, *Z =* 7.30, *P <* 0.001), which indicated that the increase in motivation with increasing reward was lower in the HD group than controls (χ^2^ = 8.40, *P =* 0.004).

The only remaining significant interaction was independent of Group (remaining Group interactions, *|Z|* < 1.67, *P* > 0.10). Specifically, the Domain × Effort × Reward interaction was significant (β = 0.74 ± 0.29, *Z =* 2.57, *P =* 0.01) and simply indicated that participants were more motivated to engage in the cognitive versus physical effort tasks at the highest level of effort (Level 6) at the lowest rewards (Levels 2–3; Supplementary Fig. 1). This essentially represents greater effort discounting for the physical than the cognitive task at lower reward levels—a finding that has been demonstrated previously.^46^

#### Controlling for reinforcement rate

Next, we considered the possibility that the lower motivation in controls versus HD may have been driven, not by the decrement in performance *per se*, but by a reduced capacity to perform each task to the reward threshold. To test for this possibility, we implemented a similar generalized linear mixed effects model, but substituted performance with the reinforcement rate for the more effortful option. This analysis provided similar model fits to the preceding analysis [improved fit by 2.1 Akaike Information Criterion (AIC) or Bayesian Information Criterion (BIC) units] and revealed a very similar pattern of simple effects and interactions compared to the preceding analysis.

Specifically, the Group × Domain interaction was again significant (β = 2.36 ± 1.00, *Z =* 2.37, *P =* 0.018) and showed that the HD group were clearly less cognitively motivated than controls (χ^2^ = 12.6, *P =* 0.0008), with group differences in the physical domain of borderline significance (χ^2^ = 3.70, *P =* 0.054; Supplementary Table 2). The Group × Effort and Group × Reward interactions were again significant, in the same pattern as described previously (Group × Effort, β = 1.28 ± 0.46, *Z =* 2.76, *P =* 0.006; Group × Reward, β = −2.32 ± 0.72, *Z =* −3.22, *P =* 0.001). The only other significant interaction was independent of Group [Domain × Effort × Reward interaction (β = 0.63 ± 0.27, Z = 2.35, *P =* 0.019; higher order Group interactions, *|Z|* < 1.52, *P* > 0.12; remaining interactions, *|Z|* ≤ 1.73, *P* > 0.08)]. An analogous model incorporating mean reinforcement rates for each participant (instead of the reinforcement rate for the more effortful option) revealed the same pattern of results (Supplementary Table 3).

In sum, these results indicate that patients with HD were clearly less willing to invest cognitive effort than controls, even after accounting for their capacity to perform each level of effort. In the physical domain, however, the lower motivation in the HD group relative to controls was weaker and only borderline significant when controlling for the capacity of participants to perform the task. Notably, this occurred despite the HD group being more impaired than controls when performing the physical effort task.

### Subjective perception of task load

**Figure 4:**
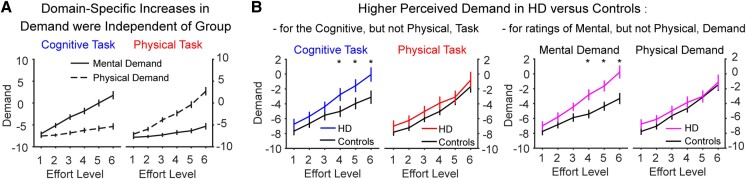
**Responses on the mental and physical demand subscales of the NASA Task Load Index.** (**A**) Data confirmed that higher levels of cognitive effort (*left*) were associated with increasing mental demand (solid lines), but not physical demand (dotted lines), while the converse was true of the physical effort task (*right*). This effect was independent of group. (**B**) Significant group differences in demand ratings. *Left*: The Huntington’s disease (HD) group perceived higher levels of the cognitive task (blue), but not the physical task (red), to be more demanding than controls (black). *Right*: The HD group (purple) also rated the mental demand, but not the physical demand, as higher than controls (black) across both tasks. Group means are plotted ± 1 standard error of the mean (SEM). *P ≤ 0.05.

To confirm that the cognitive and physical tasks effectively manipulated the subjective perception of effort in their respective domains, we analysed responses on the mental and physical demand subscales of the NASA Task Load Index. Specifically, we analysed ratings with a four-way repeated-measures ANOVA, probing for task-specific effects (cognitive, physical) at each Level (1–6) of each subscale (‘mental demand’, ‘physical demand’) as a function of Group (HD, control) (Fig. 4).

As expected, the interaction between Task × Subscale × Level was significant [*F*(1.51,56) = 44.7, *P* < 0.001; Task × Subscale, *F*(1,37) = 71.1, *P* < 0.001; Task × Level, *F*(2.3,84.5) = 1.75, *P* = 0.18; Subscale × Level, *F*(2.3,86.1) = 0.63, *P* = 0.56; Fig. 4A]. This confirmed a domain-specific effect of the cognitive and physical tasks on their corresponding subscales. Specifically, in the cognitive task, successive increases in the level of cognitive effort were accompanied by consistent increases in mental demand (all *P*-values < 0.001), but not physical demand. Conversely, on the physical task, successive increases in the level of physical effort were accompanied by consistent increases in physical demand (all *P*-values ≤ 0.003), but not mental demand. This confirms the efficacy of both tasks in manipulating the perceived demand in their corresponding domain.

In addition, there were two significant three-way interactions involving Group (Fig. 4B). First, the Group × Task × Level interaction was significant [*F*(2.3,84.5) = 3.0, *P* = 0.049; Group × Level, *F*(1.6,58.4) = 0.90, *P* = 0.39; Group × Task, *F*(1,37) = 0.94, *P* = 0.34; Fig. 4B, left panel] and was driven by higher overall demand ratings for HD versus controls at Levels 4–6 of the cognitive effort task (*P* ≤ 0.05). However, there were no group differences at any level of the physical effort task (*P*-values ≥ 0.21). Second, there was a significant Group × Subscale × Level interaction [Group × Subscale × Level, *F*(2.3,86.1) = 9.1, *P* < 0.001; Group × Subscale, *F*(1,37) = 8.7, *P* = 0.005; Fig. 4B, right panel], which revealed higher task load ratings in HD versus controls at Levels 4–6 of the mental demand subscale (Levels 4–6, *P* ≤ 0.007). Again, however, there were no group differences at any level of the physical demand subscale (all *P*-values > 0.20). Neither the three-way interaction involving Group × Task × Subscale, nor the four-way interaction, were significant [Group × Task × Subscale, *F*(1,37) = 0.001, *P* = 0.98; four-way, *F*(1.51,56.0) = 0.34, *P* = 0.64].

In summary, these results confirmed that the cognitive and physical effort tasks were effective in selectively manipulating effort within their corresponding domains. Furthermore, they showed that: (i) the HD group rated the cognitive effort task as overall more demanding than controls at higher levels of effort, but the physical effort task was rated similarly between groups; and (ii) the HD group rated the higher levels of effort across both tasks as more mentally demanding than controls, but physical demand ratings were similar between groups.

### The relationship between motivation and disease characteristics

Finally, we considered whether motivation in the HD group was related to disease characteristics—including overall cognitive function (MoCA scores), trait apathy (DAS or AES scores) and disease severity [Disease Burden Score (DBS) or Total Functional Capacity (TFC) scores]. We used mean acceptance rates as a summary measure of the overall willingness to invest effort, as has been used in previous studies.^25,27,28,47^ However, we found no significant correlations between mean acceptance rates in the HD group (in either the cognitive or physical domain, or when collapsed across both) and scores on the MoCA (*P* ≥ 0.12), the apathy scales (DAS, *P* ≥ 0.11; AES, *P* ≥ 0.41) or the disease severity scales (DBS, *P* ≥ 0.44; TFC, *P* ≥ 0.17).

## Discussion

This study aimed to determine whether the reduction in goal-directed behaviour in HD disproportionately affects one domain over another. In keeping with our previous work in pre-manifest HD,^28^ we found that participants with manifest HD were less willing than controls to engage in cognitively demanding activity, even after controlling for their impaired performance. In contrast, the HD group had similar motivation to exert physical effort as controls, despite being more physically impaired on the task and being aware of their performance through explicit feedback on each trial. Finally, this dissociation in domain-specific motivation was accompanied by differences in how the groups perceived the subjective task load of cognitive and physically effortful behaviours. Together, these data provide evidence for distinct effects of manifest HD on the willingness to exert cognitive and physical effort.

The finding that cognitive motivation was impaired in manifest HD replicates our earlier finding in those with pre-manifest disease.^28^ Importantly, this earlier study compared participants with pre-manifest HD to well matched controls who were equally capable of performing the cognitive effort task.^28^ Here, we extend this finding to a group with manifest HD, who were more cognitively impaired than controls—both on general neuropsychological tests, as well as on the cognitive effort task itself. Our data showed that the lower willingness to invest cognitive effort in HD versus controls was accompanied by greater overall demand ratings in HD versus controls at higher levels of the cognitive effort task. Importantly, however, the group differences in cognitive motivation persisted, even after accounting for differences in cognitive capacity. This finding suggests that HD is associated with a heightened sensitivity to cognitive effort and is consistent with previous work indicating greater aversion to cognitive effort in cases of striatal dysfunction.^27,28,48^

In contrast, patients with manifest HD had similar levels of physical motivation compared to controls. This again replicates our earlier finding in individuals with pre-manifest disease, who were compared to a physically well matched control group.^28^ Importantly, however, the HD group in the current study were significantly more impaired than controls—both generally (e.g. on measures of tapping) and on the physical effort task itself. Notably, all participants were aware of their performance through explicit feedback on every trial of the reinforcement phase. Despite their impairment, both groups rated the physical effort task as overall similarly demanding. It may be argued that this reflects a tendency in HD to underestimate the amount of effort involved in a motor action^49^ and/or features of our design that attenuated the perceived demand of the physical task in the HD group—for example, individuals with HD tend to benefit from real-time feedback of their performance,^50,51^ which in our study was available in the physical (as live displays of force output), but not the cognitive, effort task.

Importantly, however, these considerations may not entirely account for the seemingly paradoxical finding that the HD group were as physically motivated as controls, despite being significantly more physically impaired. Instead, this raises the possibility that HD participants were less able to recognize the extent of their physical deficits, despite receiving feedback on their consequences. Similar findings have been reported in HD in the context of anosognosia, which some data suggest may be domain-specific.^18^ For example, those with early HD may lack insight into their motor symptoms,^52^ but have relatively preserved or even heightened awareness of their cognitive symptoms.^53^ A greater loss of insight into one’s physical versus cognitive deficits could also account for the similar physical demand ratings, but higher mental demand ratings, in HD versus controls on our tasks. Of course, future work will need to confirm whether it is indeed a domain-specific lack of insight into motor disability that drives the motivation to exert physical effort regardless of an impaired ability to do so in HD.

In the context of the broader literature on motivation, these results are in keeping with the suggestion that separable mechanisms underlie motivation across different domains of effort.^23,28,44^ Previous work has implicated the striatum in motivation across the cognitive and physical domains.^24,46,54-56^ In particular, patients with other striatal disorders, such as Parkinson’s disease, have deficits in cognitive and physical motivation that are ameliorated with dopaminergic therapy.^26,27^ However, there is also accumulating data that cognitive and physical motivation are dissociable and may be driven by partially separable neurobiological mechanisms.^23,28^ The present study complements this earlier work and emphasizes the need to consider the multidimensionality of motivation and its impairments.

In this study, we found no strong associations between motivation (in terms of either effort preference or trait apathy) and disease severity. This could potentially be due to the sample size of our HD cohort. Several studies have suggested that the incidence of apathy increases with disease progression^9,10,13,57,58^ and that the severity of apathy is correlated with poorer motor function in HD.^59-61^ Notably, however, this relationship is not universal and several studies have reported the absence of such an effect.^62-64^ The reasons for such discrepancies are likely multi-factorial and may relate to varying sample size and the wide range of tools that have been used to assess apathy.^65^ This emphasizes the need for future well powered studies, ideally with the same participants followed-up longitudinally with harmonized assessments, to clarify how deficits in effort-based processing evolve with disease progression.

In conclusion, our data indicate a dissociable pattern of motivational impairments in HD. In the cognitive domain, lower motivation in HD was accompanied by a heightened perception of effort and was independent of performance capacity. In contrast, physical motivation was similar across both groups, despite the HD group experiencing a clear performance decrement relative to controls. Such maladaptive responses, in which patients have apparently ‘preserved’ levels of motivation despite increasing physical disability, have critical implications for the management of patients with HD. For example, being willing to embark on physically demanding activities that are not accomplishable may clearly lead to deleterious outcomes. More broadly, these findings emphasize that the willingness to invest effort is the final manifestation of a complex set of pathways involved in effort processing, which can be differentially impacted across separate domains of effort.

## Supplementary Material

awae023_Supplementary_Data

## Data Availability

The data that support the findings of this study are available from the corresponding author, upon reasonable request.
