# Supplementary material for: The effect of Huntington’s disease on cognitive and physical motivation
Source: Brain. 2024 Jan 24;147(7):2449–58. doi: 10.1093/brain/awae023 (PMC11224606; doi:10.1093/brain/awae023)
Supplement: awae023_Supplementary_Data [file awae023_supplementary_data.pdf]

The effect of Huntington's disease on cognitive and physical motivation

Supplementary Materials

Kelly J. Atkins[1], Sophie C. Andrews [1,2], Julie C. Stout [1], & Trevor T.-J. Chong [1,3,4,*]

[1] Turner Institute for Brain and Mental Health, School of Psychological Sciences, Monash University, Victoria 3800 Australia

[2] Thompson Institute, University of the Sunshine Coast, Queensland 4575, Australia

[3] Department of Neurology, Alfred Health, Melbourne, Victoria 3004, Australia

[4] Department of Clinical Neurosciences, St Vincent's Hospital, Melbourne, Victoria 3065, Australia

1. Details of the Primary Analyses

Here, we provide details of the primary analyses on choice behaviour. We summarise the outputs of the generalised linear mixed-effects models controlling for the effects of Performance (*Supplementary Table S1*) and Reinforcement Rate (*Supplementary Table S2*) of the more effortful option on each trial. We also conducted an additional analysis in which we entered the Mean Reinforcement Rate for each participant across all six effort levels of each task as a fixed effect (*Supplementary Table S3*). All of these analyses indicate a similar pattern of results, as summarised in the main text. Finally, we illustrate the significant Domain x Effort x Reward interaction seen across both analyses (*Supplementary Figure S1*).

Supplementary Table S1. Output of the mixed-effects model on *Choice ~ 1 + Group * Domain * Effort * Reward + Performance + (Domain + Effort + Reward + Performance | Participant)*

Fixed Effects	β	*SE*	*Z*	*p*	Sig.
(Intercept)	6.5122	0.8453	7.704	1.32E-14	*
Group	-4.2705	1.1444	-3.732	0.00019	*
Domain	-3.4915	0.8214	-4.25	2.13E-05	*
Effort	-2.6166	0.3854	-6.79	1.12E-11	*
Reward	4.0289	0.5362	7.514	5.74E-14	*
Performance	0.7184	0.2097	3.426	0.000613	*
Group x Domain	2.9914	1.1292	2.649	0.008069	*
Group x Effort	1.4003	0.4725	2.964	0.003039	*
Domain x Effort	0.2618	0.3207	0.817	0.414204	
Group x Reward	-2.2736	0.7166	-3.173	0.001509	*
Domain x Reward	-0.7173	0.3588	-1.999	0.045624	*
Effort x Reward	-0.2657	0.2454	-1.083	0.278912	
Group x Domain x Effort	-0.3014	0.3525	-0.855	0.392533	
Group x Domain x Reward	0.6618	0.3884	1.704	0.088362	
Group x Effort x Reward	0.4398	0.2692	1.633	0.102378	
Domain x Effort x Reward	0.7349	0.2747	2.676	0.00746	*
Group x Domain x Effort x Reward	-0.5443	0.3146	-1.73	0.083592	

Supplementary Table S2. Output of the mixed-effects model on *Choice ~ 1 + Group * Domain * Effort * Reward + Reinforcement + (Domain + Effort + Reward + Reinforcement| Participant)*

Fixed Effects	β	*SE*	*Z*	*p*	Sig.
(Intercept)	6.6886	0.8835	7.57	3.73E-14	*
Group	-4.2127	1.186	-3.552	3.82E-04	*
Domain	-3.1569	0.7398	-4.267	1.98E-05	*
Effort	-2.6803	0.3788	-7.075	1.49E-12	*
Reward	4.0485	0.54	7.496	6.55E-14	*
Reinforcement	0.5203	0.2585	2.013	0.044142	*
Group x Domain	2.3647	0.997	2.372	0.017703	*
Group x Effort	1.2837	0.4647	2.762	0.005739	*
Domain x Effort	0.1673	0.3084	0.542	0.587536	
Group x Reward	-2.321	0.7207	-3.221	0.001279	*
Domain x Reward	-0.6019	0.3475	-1.732	0.083259	
Effort x Reward	-0.2137	0.2364	-0.904	0.365891	
Group x Domain x Effort	-0.2228	0.3411	-0.653	0.513611	
Group x Domain x Reward	0.5712	0.3781	1.511	0.130873	
Group x Effort x Reward	0.3948	0.2603	1.517	0.129322	
Domain x Effort x Reward	0.6255	0.2665	2.347	0.018914	*
Group x Domain x Effort x Reward	-0.445	0.3062	-1.453	0.146196	

Supplementary Table S3. Controlling for Mean Reinforcement Rates for each Participant. Output of the mixed-effects model on *Choice ~ 1 + Group * Domain * Effort * Reward + Reinforcement + (Domain + Effort + Reward | Participant)*

Fixed Effects	β	SE	Z	p	
(Intercept)	6.7869	0.9412	7.211	5.56E-13	*
Group	-4.4266	1.3083	-3.384	0.000716	*
Domain	-3.2597	0.7546	-4.32	1.56E-05	*
Effort	-2.7468	0.3914	-7.018	2.25E-12	*
Reward	4.0646	0.544	7.471	7.93E-14	*
Reinforcement	0.6563	0.2639	2.487	0.012899	*
Group x Domain	2.7421	1.0364	2.646	0.008148	*
Group x Effort	1.2821	0.4906	2.613	0.008966	*
Domain x Effort	0.1813	0.3082	0.588	0.556406	
Group x Reward	-2.3287	0.7263	-3.206	0.001344	*
Domain x Reward	-0.605	0.3482	-1.737	0.082331	
Effort x Reward	-0.2327	0.2357	-0.987	0.323501	
Group x Domain x Effort	-0.2155	0.3389	-0.636	0.524866	
Group x Domain x Reward	0.5224	0.3787	1.379	0.167745	
Group x Effort x Reward	0.435	0.2597	1.675	0.093898	
Domain x Effort x Reward	0.6246	0.2664	2.344	0.019066	*
Group x Domain x Effort x Reward	-0.5113	0.3054	-1.674	0.094143	

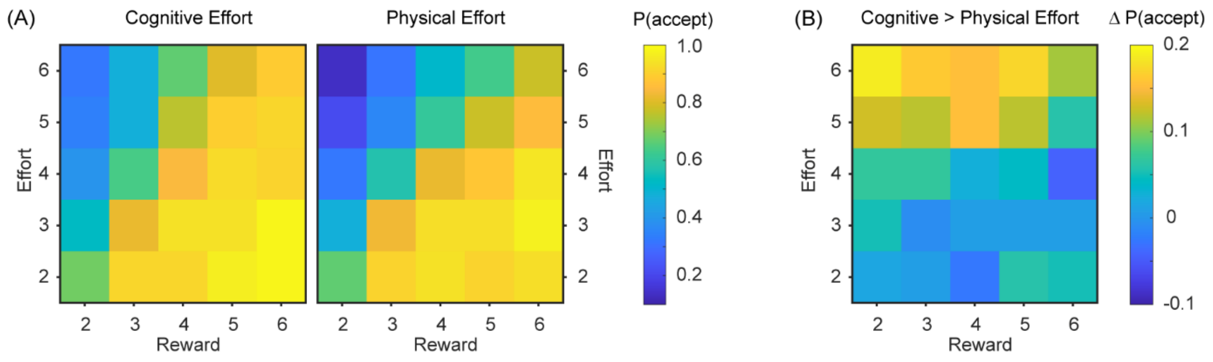

Supplementary Figure S1. *Domain x Effort x Reward interaction.* (A) The probability of accepting the more effortful option as a function of Effort and Reward (*P(accept)*), plotted separately for the cognitive (left) and physical (right) effort tasks. Yellow = more motivated, blue = less motivated. (B) Difference plot indicating greater cognitive vs physical motivation. Each cell represents the difference in *P(accept)* between the cognitive and physical tasks. Yellow = more cognitively motivated; blue = more physically motivated. Individuals were more cognitively than physically motivated at the highest level of effort (level 6) at the lower levels of reward (levels 2-3). The interaction with Group was not significant.

2. Effects of Disease Characteristics

An important aspect of our analyses was to control for those features that were likely to impact behaviour on our task. Specifically, as detailed in the primary analyses, it was critical to control for the capacity of individuals to perform each task, in order to ensure that any aversion to effort was due to low motivation, rather than an inability to perform those levels in the first place. Thus, in the primary analyses on choice preference, we performed separate analyses controlling for the effects of both Performance and Reinforcement Rate on the willingness to exert effort.

Although *Table 1* highlights several demographic features that differed between the HD and control groups, we did not include these features as additional fixed effects, given that many of these factors: (1) provided broader, less specific measures of performance on our task (e.g., controlling for generic cognitive capacity with MoCA scores is less precise than using target detection sensitivity (*d'*) unique to each level of effort on our task); and (2) were collinear with those already included in our model (e.g., the correlation between MoCA scores and *d'* was highly significant; Spearman's $\rho = 0.63$, $p = .000016$). Furthermore, supplementary analyses substituting some of these more generic measures for the more specific fixed effects of Performance / Reinforcement Rate reveal the same pattern of results as in the primary analyses (e.g., substituting MoCA as a fixed effect, *Supplementary Table S4*).

Finally, we note that we did not include apathy scores (on the DAS or AES) in our primary analyses to avoid circularity. Many recent studies have argued that a lower willingness to exert effort is a core component of apathy (e.g., Lafond-Brina et al. (2023). *Cerebral Cortex*; Le Bouc et al. (2023). *Brain*; Saleh et al. (2021). *Brain*; Le Heron et al. (2018). *Brain*; Lockwood et al. (2017). *Nature Human Behaviour*). Indeed, we have shown that effort discounting on our tasks is highly correlated with scores on the DAS (Jurgelis et al. (2021). *Scientific Reports*). Including the DAS score into our mixed-effects models would therefore have been circular – in effect, asking whether the HD and control groups differed in their willingness to exert effort (choice data), after controlling for their willingness to do so in the first place (DAS scores).

Supplementary Table S4. Controlling for MoCA. Output of the mixed-effects model on *Choice ~ 1 + Group * Domain * Effort * Reward + MoCA + (Domain + Effort + Reward | Participant)*

Fixed Effects	β	*SE*	*Z*	*p*	Sig.
(Intercept)	6.5085	0.928	7.013	2.33E-12	*
Group	-4.5532	1.3608	-3.346	0.00082	*
Domain	-3.2543	0.8554	-3.804	0.000142	*
Effort	-2.6259	0.4065	-6.46	1.05E-10	*
Reward	3.9464	0.5543	7.12	1.08E-12	*
MoCA	0.5211	0.575	0.906	0.364791	
Group x Domain	3.2011	1.1717	2.732	0.006295	*
Group x Effort	1.2179	0.5001	2.435	0.014878	*
Domain x Effort	0.1562	0.3267	0.478	0.632526	
Group x Reward	-2.2083	0.7301	-3.025	0.002489	*
Domain x Reward	-0.6123	0.3713	-1.649	0.099127	
Effort x Reward	-0.1735	0.2489	-0.697	0.485851	
Group x Domain x Effort	-0.1705	0.3567	-0.478	0.632613	
Group x Domain x Reward	0.5159	0.4015	1.285	0.198847	
Group x Effort x Reward	0.3853	0.2728	1.412	0.15791	
Domain x Effort x Reward	0.6247	0.2794	2.236	0.025373	*
Group x Domain x Effort x Reward	-0.5211	0.3188	-1.634	0.102171	

3. NASA Task Load Index

In the main manuscript, we analyse responses on domain-specific subscales of the NASA Task Load Index (Mental Demand, Physical Demand). The entire NASA Task Load Index comprises four other subscales, which measure the perception of Temporal Demand ('How hurried or rushed was the pace of the task?'), Effort ('How hard did you have to work to accomplish your level of performance?'), Performance ('How successful were you in accomplishing what you were asked to do?'), and Frustration ('How insecure, discouraged, irritated, stressed, and annoyed were you?'). [43] Higher scores indicated higher perceived task load (i.e., *greater* mental demand, physical demand, temporal demand, effort, and frustration; and *lower* perceived performance). Here, we provide a summary of participant responses on the Temporal Demand, Effort, Performance and Frustration subscales. We analysed responses on each subscale with a three-way Group (HD, controls) x Task (cognitive, physical) x Level (1-6) repeated measures ANOVA (***Supplementary Figure S2***).

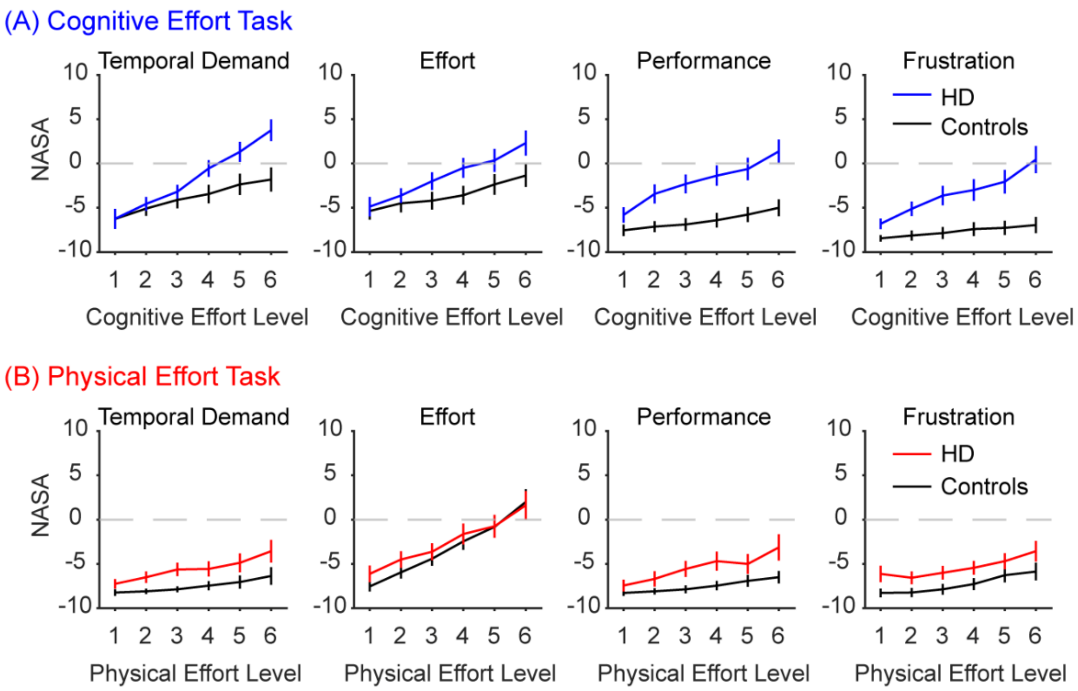

Supplementary Figure S2. Data from the NASA Task Load Index for the (A) Cognitive Effort Task, and (B) Physical Effort Task, plotted separately for HD (blue/red) and controls (black). In addition to their perceived mental and physical demand (reported in the main text), participants rated the temporal demand, overall effort, level of performance, and frustration that they experienced at each level of each task. The NASA Task Load Index is a 21-point scale (range -10 to 10). Note that higher scores on the 'Performance' subscale indicate *poorer* perceived performance. Mean values are plotted ± 1 SEM.

Temporal Demand. Analysis of the Temporal Demand subscale revealed significant two-way interactions between Group x Level, and Task x Level (Group x Level, $F(1.9, 70.3) = 5.0$, $p = .01$; Task x Level; $F(2.08, 76.8) = 12.7$, $p < .001$; Group, $F(1,37) = 6.10$, $p = .018$; Task, $F(1, 37) = 35.2$, $p < .001$; Level, $F(1.9, 70.3) = 37.6$, $p < .001$). The Group x Level interaction indicated that the HD group rated higher levels of the tasks as more temporally demanding than controls (Levels 1-3, $p \geq .07$; Levels 4-6, $p \leq .015$). The Task x Level interaction indicated that temporal demand increased more consistently across successive levels of the cognitive effort task (between effort levels 2-3, 3-4 and 4-5, $p \leq .03$; level 5-6, $p = .086$), but only increased between levels 5-6 of the physical effort task ($p = .029$, else $p \geq .16$). The Group x Task interaction was not significant (Group x Task, $F(1, 37) = 3.063$, $p = .80$). The three-way interaction was of borderline significance ($F(2.08, 76.8) = 2.92$, $p = .06$), and appeared to be driven by the HD group perceiving all levels to be more temporally demanding than controls ($p \leq .025$), other than at the lowest level of physical effort ($p = .3$).

Effort. Analysis of general Effort ratings revealed a significant three-way interaction (Group, $F(1,37) = 1.63$, $p = .21$; Task, $F(1, 37) = 0.26$, $p = .62$; Level, $F(1.53, 56.5) = 53.9$, $p < .001$; Group x Task, $F(1, 37) = 1.01$, $p = .32$; Task x Level, $F(2.35, 87.1) = 4.81$, $p = .007$; Group x Level, $F(1.53, 56.5) = 0.25$, $p = .72$; three-way, $F(2.35, 87.1) = 3.64$, $p = .024$). Decomposing this interaction revealed that, in both groups, progressive increases in levels of the physical task were accompanied by higher ratings of overall effort. However, progressive increases in levels of the cognitive task were associated with steeper increases in effort ratings in the HD group relative to controls. This suggests that the HD group were more sensitive to increases in cognitive effort than controls, but similar in their ratings of physical effort.

Performance. The main effect of Task was significant, indicating higher Performance ratings in the cognitive relative to the physical effort task (Task, $F(1, 37) = 11.8$, $p < .001$). The main effects of Group and Level were significant, and qualified by a significant two-way interaction (Group, $F(1,37) = 14.9$, $p < .001$; Level, $F(2.65, 98.2) = 34.7$, $p < .001$; Group x Level, $F(2.65, 98.2) = 6.78$, $p < .001$). This indicated that the HD group provided higher Performance ratings than controls for all levels of effort, other than the lowest (level 1, $p = .076$; Levels 2-6, $p \leq .005$). No other interactions were significant (Group x Task, $F(1, 37) = 3.24$, $p = .08$; Task x Level, $F(2.14, 79.2) = 2.18$, $p = .12$; three-way, $F(2.14, 79.2) = 0.90$, p

= .42). This suggests that the HD group generally rated their performance as poorer than controls (other than at the lowest level of effort).

Frustration. Finally, analysis of frustration ratings revealed a significant three-way interaction (Group, $F(1, 37) = 15.3$, $p < .001$; Task, $F(1, 37) = 1.38$, $p = .25$; Level, $F(2.12, 78.4) = 27.3$, $p < .001$; Group x Task, $F(1, 37) = 2.99$, $p = .09$; Group x Level, $F(2.12, 78.4) = 4.36$, $p = .014$; Task x Level, $F(1.99, 73.8) = 2.93$, $p = .06$; three-way, $F(1.99, 73.8) = 6.04$, $p = .004$). This interaction revealed that the HD group provided higher frustration ratings than controls at all levels of the cognitive effort task ($p \leq .02$), but only at the lowest level of the physical effort task ($p = .036$; else $p \geq .06$). This suggests that the HD group were more frustrated than controls in the cognitive task, but group differences were less pronounced in the physical task.

---

4. References

Lafond-Brina G, Pham BT, & Bonnefond A. Initiative apathy trait underlies individual differences in the ability to anticipate and expend cognitive effort in cost-benefit decision-making tasks. *Cerebral Cortex*. 2023; 33(12), 7714-7726.

Le Bouc R, Borderies N, Carle G, *et al*. Effort avoidance as a core mechanism of apathy in frontotemporal dementia. *Brain*. 2023; 146(2), 712-726.

Le Heron C, Plant O, Manohar S, *et al*. Distinct effects of apathy and dopamine on effort-based decision-making in Parkinson's disease. *Brain*. 2018;141(5):1455-1469.

Lockwood PL, Hamonet M, Zhang SH, *et al*. Prosocial apathy for helping others when effort is required. *Nature Human Behaviour*. 2017; 1(7), 131.

Saleh Y, Le Heron C, Petitet P, *et al*. Apathy in small vessel cerebrovascular disease is associated with deficits in effort-based decision making. *Brain*. 2021; 144(4), 1247-1262.
